# Combining adaptive expertise and (critically) reflective practice to support the development of knowledge, skill, and society

**DOI:** 10.1007/s10459-022-10178-8

**Published:** 2022-11-09

**Authors:** Stella L Ng, Jacquelin Forsey, Victoria A Boyd, Farah Friesen, Sylvia Langlois, Kori Ladonna, Maria Mylopoulos, Naomi Steenhof

**Affiliations:** 1 Centre for Advancing Collaborative Healthcare and Education, University of Toronto, Toronto, Canada; 2 Rehabilitation Sciences Institute, University of Toronto, Toronto, Canada; 3 Institute of Health Policy, Management and Evaluation, University of Toronto, Toronto, Canada; 4 Temerty Faculty of Medicine, Toronto, Canada; 5 Department of Innovation in Medical Education, University of Ottawa, Ottawa, Canada; 6 The Wilson Centre, Temerty Faculty of Medicine, University of Toronto, Toronto, Canada; 7 Leslie Dan Faculty of Pharmacy, University of Toronto, Toronto, Canada

**Keywords:** Adaptive expertise, Reflective practice, Critical reflection, Pedagogy, Interprofessional

## Abstract

Adaptive expertise (AE) and reflective practice (RP), two influential and resonant theories of professional expertise and practice in their own right, may further benefit health professions education if carefully combined. The current societal and systemic context is primed for both AE and RP. Both bodies of work position practitioners as agentive, learning continually and thoughtfully throughout their careers, particularly in order to manage unprecedented situations well. Similar on the surface, the roots and practices of AE and RP diverge at key junctures and we will focus on RP’s movement toward *critically* reflective practice. The roots of AE and RP, and how they relate to or diverge from present-day applications matter because in health professions education, as in all education, paradigmatic mixing should be undertaken purposefully. This paper will explore the need for AE and RP, their shared commitments, distinctive histories, pedagogical possibilities both individually and combined, and next steps for maximizing their potential to positively impact the field. We argue that this exploration is urgently needed because both AE and RP hold much promise for improving health care and yet employing them optimally—whether alone or together—requires understanding and intent. We build an interprofessional education case situated in long-term care, throughout the paper, to demonstrate the potential that AE and RP might offer to health professions education individually and combined. This exploration comes just in time. Within the realities of uncertain practice emphasized by the pandemic, practitioners were also called to act in response to complex and urgent social movements. A combined AE and RP approach, with focus on critically reflective practice in particular, would potentially prepare professionals to respond effectively, compassionately, and equitably to future health and social crises and challenges.

Adaptive expertise (AE) and reflective practice (RP), two influential and resonant theories of professional expertise and practice in their own right (AMEE 2018; Eraut 1994; Fragkos 2016; Hammerness et al. 2005) may further benefit health professions education if carefully combined. Consider these two definitions. Through adaptive expertise, “…as clinicians work, particularly in situations of novelty and complexity, they often find that straightforward applications of their knowledge are insufficient to address patient needs. Instead, they are required to use their knowledge flexibly to develop an effective solution within the patient, social, and system contexts in which they find themselves” (Mylopoulos et al. 2016). Meanwhile, “The [reflective] practitioner allows [themselves] to experience surprise, puzzlement, or confusion in a situation which [they] find uncertain or unique. [They] reflect on the phenomena before [them] and on the prior understandings which have been implicit in [their] behaviour. [They] carry out an experiment which serves to generate both a new understanding of the phenomena and a change in the situation” (Schön 1983). Perhaps now more than ever, AE and RP offer much-needed hope to the health professions, hard-hit by the global pandemic. The pandemic has shaken the health care system, disrupting scopes of practice and well-established roles, rapidly shifting models of care, and deploying students as externs and residents as leaders ahead of usual timelines (Aruru et al. 2021; Benton et al. 2021; Bosveld et al. 2021; Flotte et al. 2020; Leslie et al. 2021; Marani et al. 2021; McGilton et al. 2021; Seneviratne et al. 2020; Soled et al. 2020; Stucky et al. 2021; Uchida et al. 2020). Meanwhile, patient needs must be met in a context of social unrest and distrust in health care institutions (Balog-Way and McComas 2020; Estabrooks et al. 2020; Gisondi et al. 2022; Graham 2022; Phillips-Beck et al. 2020; Proof Strategies 2022; Smith et al. 2021).

This societal and systemic context is primed for both AE and RP, and perhaps necessitates their thoughtful integration into health professions education and practice. Both bodies of work position practitioners as agentive, learning continually and thoughtfully throughout their careers, particularly in order to manage unprecedented situations well. Professionals mobilize their adaptive expertise to handle ambiguity, complexity, and uncertainty (Mylopoulos et al. 2018). They engage in reflective practice to navigate indeterminate zones of practice, defined as uncertain, unstable, unique, and value-conflicted (Schön 1983, 1987). Post-pandemic, new, uncertain challenges will arise. Whether these challenges include an environmental crisis, the health human resource crisis, continuing social turmoil, or a combination of these, AE and RP can help prepare learners for these future challenges now.

Similar on the surface, the roots and practices of AE and RP diverge at key junctures (see Table 1). These roots and how they relate to or diverge from present-day applications matter because in health professions education, as in all education, paradigmatic mixing should be undertaken purposefully. Education paradigms represent underlying assumptions about the purpose of education, the roles and goals of teachers and learners, and the teaching and assessment approaches best suited to achieve these purposes and goals (Baker et al. 2019, 2021). When paradigms and practices align or are mixed deliberately (Baker et al. 2019, 2021), education may achieve epistemological coherence during the learning experience (Ng et al. 2019a) reinforced through compatible assessment methods (Tavares et al. 2020, 2021). Conversely, when the philosophical roots of an educational approach are neglected, practices may misalign, and students may experience a sense of incongruence in their learning and incompatibility in their assessment. For example, reflective assignments in the health professions often over-instrumentalize the process, leading to reflection fatigue, inauthentic reflection, and perceived surveillance (de la Croix and Veen 2018; Hodges 2015; Nelson and Purkis 2004; Ng et al. 2015). Thus, we would recommend that in combining and applying AE and RP, efforts should be mindful of history, theory, and purpose. This paper will explore the need for AE and RP, their shared commitments, distinctive histories, pedagogical possibilities both individually and combined, and next steps for maximizing their potential to positively impact the field. We argue that this exploration is urgently needed because both AE and RP hold much promise for improving health care and yet employing them optimally—whether alone or together—requires understanding and intent.

We will build on the following interprofessional case/story, situated in long-term care, throughout the paper to demonstrate the potential that AE and RP might offer to health professions education.

Samuel is an 85-year-old man with a close-knit family and a penchant for live music. He has been diagnosed with early-stage Alzheimer’s Disease and has workplace-acquired hearing loss after working for many years at an automotive manufacturing facility. In the past, Samuel has experienced delirium, which warranted a re-evaluation of his medications. He has recently experienced an extended hospitalization due to complications of COVID-19. While in hospital, Samuel’s cognitive and physical status declined, resulting in reduced ability to manage his self-care independently. Samuel has enjoyed daily conversations with his wife, whom he lives with in a suburban house, and weekly conversations with his son and grandchildren. The interprofessional team is now discussing discharge to a long-term care setting, and there are many different needs and perspectives to consider in doing so.

**Table 1:** Key points on adaptive expertise and reflective practice

	Adaptive expertise	Reflective practice
Definition	“…as clinicians work, particularly in situations of novelty and complexity, they often find that straightforward applications of their knowledge are insufficient to address patient needs. Instead, they are required to use their knowledge flexibly to develop an effective solution within the patient, social and system contexts in which they find themselves.” (Mylopoulos et al. 2016)	“The practitioner allows [themself] to experience surprise, puzzlement, or confusion in a situation which [they] find uncertain or unique. [They] reflect on the phenomena before [them] and on the prior understandings which have been implicit in [their] behaviour. [They] carry out an experiment which serves to generate both a new understanding of the phenomena and a change in the situation.” (Schön 1983)
Foundational theorists	• Giyoo Hatano and Kayoko Inagaki (1986) • Carl Bereiter and Marlene Scardamalia (1993)	• John Dewey (1910, 1938) • Jurgen Habermas (1971) • Paulo Freire (2000) • Donald Schön (1983, 1987) • Stephen Brookfield (1998; 2000) • Michael Polanyi (1958)
Contemporary theorists in professional education	• Daniel Schwartz and John Bransford (Schwartz et al. 2005; Schwartz and Bransford 1998) • Maria Mylopoulos (Mylopoulos et al. 2018; Mylopoulos and Woods 2017)	• Elizabeth Anne Kinsella (Kinsella 2006a, 2007; Kinsella et al. 2012) • Jennifer Moon (1999, 2004) • Stella Ng (Cheng et al. 2017; Ng 2012; Ng et al. 2015, 2020, 2022).
Associated pedagogies/teaching approaches	• Integrated instruction (Kulasegaram et al. 2013) • Contrasting cases and meaningful variation (Mylopoulos et al. 2018; Schwartz and Martin 2004) • Productive failure (Kapur 2014, 2016; Mylopoulos et al. 2018)	• Arts-based or aesthetic education (Kinsella and Bidinosti 2016) • Dialogue (Boyd et al. 2022; Kumagai and Naidu 2015) • Guided reflection (Johns 2002) • Reflective writing (Bolton 2005; Moon 2004)
Variations and related constructs	• Clinical reasoning (Mylopoulos and Woods 2009) • Preparation for future learning (Mylopoulos et al. 2016; Mylopoulos and Woods 2014)	• Critical reflection (Ng et al. 2019a, 2020) • Evidence-based practice (Mantzoukas 2007) • Phronesis (Flaming 2001; Kinsella and Pitman 2012) • Praxis (Ng and Wright 2017) • Practical knowledge (Kumagai 2014;
Not to be confused with	• Master adaptive learner (Cutrer et al. 2017) • Adaptive learning (Ruiz et al. 2006)	• Self-reflection (Lew and Schmidt 2011) • Metacognition (Quirk 2006)

## The evolution of adaptive expertise

Hatano and Inagaki (1986) first conceptualized adaptive expertise in a seminal paper entitled “Two Courses of Expertise.” In this paper, the authors described various paths a learner might take on their journey from novice to expert. They positioned adaptive expertise in contrast to routine expertise. Routine expertise represents the knowledge and skills that enable efficiency to implement known routines. Adaptive expertise includes knowledge and skills that support both efficiency when working under usual circumstances, and also the capability to apply flexible problem-solving approaches and generate new solutions as the context demands.

Building on the early work by Hatano and Inagaki, contemporary theorists in the cognitive sciences, including Schwartz, Bransford, and Sears (2005), describe AE as a complementary balance between innovation and efficiency. They argued that adaptive expertise requires experts to work within an ‘optimal adaptability corridor’; they are able to efficiently apply past solutions when appropriate and generate new solutions when needed. Crucially, too much ‘innovation’ is just as problematic as too much ‘efficiency.’ The former results in unnecessarily utilizing time and resources to generate new solutions to known problems and the latter results in inappropriately trying to fit new challenges into known solutions. To ensure the development of optimal adaptability, experts must acquire procedural fluency (reproducing effective solutions), as well as conceptual understanding (knowing the rationale and mechanisms for an action or decision). Adaptive experts are then able to draw on both procedural and conceptual knowledge in their problem solving as needed. This ability frees them from being tied to a single solution in novel or complex situations, as they are able to maintain the rationale and adapt the action when necessary.

More recently, considerable research focuses on demonstrating and evolving AE as a resonant construct in health professions education and building an evidence base for various pedagogical approaches that lead to AE (Mylopoulos et al. 2012; Mylopoulos and Farhat 2015). Some key pedagogies to foster AE, aligned with cognitivist and constructivist paradigms of education (which aim for knowledge transfer and building (Baker et al. 2021)) include integrated instruction, meaningful variation, and productive failure. Integrated instruction in health professions education involves the deliberate linking of clinical concepts with basic science mechanisms during learning (Kulasegaram et al. 2013). Meaningful variation is an instructional design strategy that utilizes contrasting cases to allow learners to experience meaningful variation around key concepts (Schwartz and Martin 2004). Productive failure is a guided discovery instructional design strategy that engages students in problem solving, followed by teaching of a central concept and procedures, which develops conceptual knowledge and transfer of learning (Kapur 2014; Mylopoulos et al. 2018; Steenhof et al. 2019, 2020).

### Applying to our case

Consider our “case” of Samuel. An AE lens would recognize that learners often gravitate towards the ‘certainty’ of guidelines, confident that their management and/or discharge plan is appropriate because they matched the patient to the correct pathway in the flow diagram. However, total reliance on guidelines does not support adaptive expertise. Teaching to support adaptive expertise could include asking students to prepare a transitional care plan for Samuel without support from an instructor, and then coming together afterwards to compare and contrast their care plan to an expert’s care plan. The facilitator should be sure to highlight explicitly the context they considered (i.e. Samuel has experienced delirium prior to his COVID-19 infection, which warranted a re-evaluation of his medications). This activity utilizes both pedagogical techniques of productive failure and meaningful variation. Another strategy, supporting cognitive integration through integrated instruction, would be specifically associating the physiologic mechanisms underlying Samuel’s delirium and how these connect clinically to medications which may lower the delirium threshold. For example, the facilitator could discuss how ranitidine, a medication used to treat gastrointestinal reflux, is one potential cause for delirium. When ranitidine blocks histamine 2 receptors it can lead to diminished alertness, delayed reaction time, and somnolence, all physiological reactions that may increase Samuel's chance of becoming delirious. Tapering and either eventually discontinuing ranitidine or replacing with an alternative medication could reduce his future risk of delirium and allow for a more successful transition to long-term care.

The above example may represent more common applications of AE. To perform as adaptive experts, learners must demonstrate growing proficiency in their profession-specific management of patients. Yet they must also develop understanding of the “new” basic sciences of collaboration, communication, and team dynamics required for interactions with patients/ family/informal caregivers, and other health workers that enable a shared decision-making process (Chaudhary et al. 2019; Forsey et al. 2021; Lucey 2013). Demonstrating adaptive expertise requires intentionality and a clear understanding of everyone’s roles and responsibilities. Opportunities to wrestle with a variety of cases reflecting the complexity of interacting physical, psychological/emotional, contextual, and social factors could support the development of adaptive expertise of both individual students and collective teams of learners. Altogether, adaptive expertise may be fostered through a longitudinal interprofessional education experience that links classroom to practical learning, engages pedagogies aligned with fostering AE, and integrates the basic sciences (both traditional and “new”) and clinical science.

## The evolution of reflective practice

The history of reflective practice is somewhat more entangled, given the plethora of influences upon the construct of reflection. Reflective practice specifically begins with Schön, who studied what practitioners do in unique, uncertain, unstable, and value-conflicted moments of practice, what Schön referred to as indeterminate zones of practice. Schön argued that technical rationality alone, meaning the instrumental application of theory and technique derived from scientific knowledge, may be insufficient in these messy zones of practice. Instead, he advanced a model of professional practice that recognizes that knowledge must be continually generated by the practitioner and shaped to meet the needs of the current situation before them. Reflective practice is thus an epistemology of practice; it describes how knowledge is developed and enacted in and through professional practice. Understood as a way of knowing that is engaged continually in practice, it actively resists traditional notions of expertise and evidence that eschew uncertainty, maintain distance between expert and client, and seek deference relative to the professional. Schön wrote about shifting the typical professional-client dynamic from one of putting trust in the professional to one of joining with the professional to make sense, test judgements, and create knowledge together (Schön 1983). Schön, however, did not coin the term reflection itself. He drew heavily from Dewey’s definition of reflection as the active, persistent, and careful consideration of knowledge claims, their sources, and their implications (Dewey 1910). He linked this definition to his studies of what practitioners *do* and aimed to legitimize reflection-in-action as a form of professional knowing. Schön integrated reflection into theories of professional practice through *The Reflective Practitioner: How Professionals Think in Action* (Schön 1983). He closely studied the practice of a range of professionals, from architects to psychotherapists, and drew upon key theorists like Dewey and other constructivist thinkers (see Kinsella (2006a) for more) to build his rich description of professional practice. The concept of reflection is of course understood in many ways, and others have traced its long history (Hodges 2015; Kinsella 2012; Moon 1999; Ng 2012).

While current-day applications are often more explicit about their criticality, reflective practice has historically hinted at critical elements, as reflected in the work of scholars like Brookfield (1998; 2000), Freire (2000), Habermas (1971), and Kemmis (Carr and Kemmis 1986; Kemmis 2005). Contemporary theorists like Kinsella (Kinsella 2006b, 2012; Kinsella et al. 2012; Kinsella and Bidinosti 2016; Kinsella and Pitman 2012; Kinsella and Whiteford 2009) and Ng (Boyd et al. 2022; Ng et al. 2015, 2019b, 2020, 2022;) have continued to advance theories of both reflection and reflective practice within health professions education. These theorists have drawn out and elaborated on the critical orientation that was somewhat lacking in Schön’s theory, through a focus on critical reflection and critically reflective practice. They have also aimed to add specificity to the many definitions and uses of reflective concepts. At present, how “reflection” is used within reflective practice in health professions education runs the gamut from self-reflection to collaborative reflection (Rolfe 2014), aesthetic (Kinsella and Bidinosti 2016), embodied (Kinsella 2008), and mindfulness (Kinsella 2012) approaches to reflection, to critical reflection (Ng et al. 2019b, 2020). Whereas self-reflection may shift focus from knowledge claims and their implications toward personal knowledge and growth, critical reflection shifts focus to structural and systemic forces—including power and hierarchy—acting upon (and constraining) knowledge and practice (Kinsella 2012; Ng et al. 2019b). Yet the differentiation of these concepts is often not elucidated for HPE learners, who might then further conflate, for example, self-reflection and critical reflection. RP discussed in this paper aligns more with critical reflection than self-reflection, aesthetic reflection, embodied reflection, or mindfulness. This focus is a choice we make as authors given the apparent relevance of critically reflective practice to current social and health system needs (Halman et al. 2017; Kumagai and Lypson 2009; Ng et al. 2019b; Rowland and Kuper 2018; Sharma et al. 2018). In general, key pedagogies toward achieving/fostering *critically* reflective practice have drawn from humanistic and transformative paradigms of education, which aim to break down the barriers of the professional self, professional hierarchies, and societal inequities to enable individual, social, and systemic change (Baker et al. 2021). As such, aligned pedagogies commonly engaged to foster critically RP include arts-based approaches (Greene 1986; Kinsella and Bidinosti 2016), critical and reflexive questioning (Thille et al. 2018), and dialogic learning experiences (Boyd et al. 2022; Kumagai and Naidu 2015). Additional pedagogies for reflective practice are noted in Table 1; however we focus here on those that may hold clearest potential for critically reflective practice. Arts-based approaches invite students to represent their ethical dilemmas or responses to challenging or moving practice experiences through artistic representations, in order to unpack these with fellow learners through dialogue (Kinsella and Bidinosti 2016). Connecting more firmly to critically reflective practice, critical question-posing (such as challenging through dialogue a dominant construct in terms of who it helps and harms, and how else it could be constructed (Nixon et al. 2017)) can enhance learners’ subsequent ability to notice and talk in ways that are more equity-oriented, empathic, and collaborative (Ng et al. 2022). Dialogic learning can not only influence critically reflective ways of *seeing*, but also critically reflective ways of *doing* through subsequent actions that challenge conventional yet potentially oppressive practices (e.g. clinical letter-writing) (Boyd et al. 2022). Importantly, these pedagogies focus on maintaining space for not knowing and remaining open-ended. This approach to education is aligned with RP by allowing and encouraging diverse and multiple stories without endings, emphasizing that many complex social considerations will have no straightforward or universal solution (Kumagai et al. 2009; Kumagai and Naidu 2015). As Schön wrote, reflective practice situates uncertainty as a birthplace of learning.

### Returning to the story of Samuel

Considering the case of Samuel through a critically reflective practice orientation, first we now refer to it as a “story” in alignment with an RP epistemology. Attention would shift from knowledge integration and solutions towards how one would notice and navigate the messy, perhaps unanswerable, social dilemmas that may confront health workers in long-term care. While the same case could be used, the personal and situational details of Samuel as an individual must be foregrounded in a storied rather than more technical way and—perhaps—more questions than answers may be uncovered. Important structural and social factors would be foregrounded, such as the current staffing crisis in long-term care settings. Reduced access for family visitors for long-term care residents during a pandemic may be noticed by health workers, and may cause considerable strain, perhaps even contributing to burnout or moral distress. This reduced access may also mar relationships between health workers and family caregivers, which may lead to challenges in constructing a person- and family-centered discharge plan. An interprofessional health, arts, and humanities approach may be engaged in which learners read and discuss stories of the experiences of residents with varying complex conditions in long-term care settings where dementia and communication are declining because of staff reductions and visitor restrictions. They could engage in dialogue about their responses to similar experiences. Learners may complete a follow-up placement/practicum in long-term care, where they are asked to pay particular attention to the health system challenges and their impacts on ethical, collaborative, and compassionate care. Experienced facilitators may guide an imaginative dialogue of how a team approach might bolster morale and fill gaps, or how health system solutions could address the strain on both systems and workers. Furthermore, engaging in dialogue with family caregivers, learners from other health professions, and their peers, would enable consideration of additional perspectives. When it comes time for discharge, a critically reflective practice approach may enable the practitioners to appreciate the limitations imposed by the homecare system. Samuel might need the support of personal support workers (PSWs) who may be strapped for time. Samuel is in the fortunate position of having supportive family who are well-positioned to take time and care for him. Others have less support at home; a critically reflective practitioner would recognize this differential and, over time, aim toward greater equity in the system.

A reflective practitioner would also recognize they will always have knowledge gaps and situated perspectives. In the AE case, learners were taught about why the mechanistic action of ranitidine may influence dementia. Learners being educated for RP might focus on the fact that when discussing this, families might become (understandably) frustrated that a medication intended to help may contribute to harm. Ranitidine has been making headlines in the past few years due to higher than acceptable levels of a carcinogen (NDMA) As a learner, the reflective practitioner recognizes the limits of their own knowledge of this emerging situation and rather than responding immediately from a position of authority, makes the time to hear out the family’s concerns, validate their frustration, and work together on care plans moving forward. Following this interaction, the learner, as reflective practitioner, may debrief with the interprofessional team, including the pharmacy team, to better understand the current state of scientific evidence on ranitidine to inform future practice.

## Integrating AE and RP for better theory and pedagogy

The two examples above have been somewhat oversimplified for illustrative purposes. An AE approach could, of course, foreground moral and systemic complexities and their impact on professional well-being. An RP approach could include greater attention to the underlying relationships between medications and delirium. Yet they do not tend to. We have situated the examples in the domains of comfort and strength of each framing (AE or RP). We now shift to exploring how combining AE and RP purposefully could advance theory and pedagogy.

### Advancing theory, practice and research

It may seem surprising, at first, that AE and RP evolved as distinct traditions, given their shared goal of describing the complexity of professional expertise and practice and educating for professionals who are adept at navigating uncertainty. Part of the disconnect in the extant literature may originate in different (and often implicit) treatments of knowledge. Both bodies of work meet in history via Dewey, recognize the roles of multiple forms of knowledge, and have a somewhat subversive tone in relation to static and hierarchical positionings of knowledge and expertise. Yet they diverge in terms of where their focus eventually lands and also diverge in the conditions in which they tend to be applied. In health professions, educators have more often applied AE to curriculum design and clinical reasoning within a frame of knowledge building for medical expertise, and not on the challenging of structures of medicine itself. That said, in AE scholars Scardamalia and Bereiter’s *Surpassing Ourselves* (1993), knowledge building communities position knowledge as an assumptive artifact that can be worked on collectively. Thus proponents of AE have indeed challenged the premise of education that would position students as 'acquiring knowledge' as well as traditional notions of who can produce knowledge in society (Bereiter and Scardamalia 1993). Meanwhile, many who have continued to advance RP and its associated pedagogies have highlighted critical knowledge that has emancipatory interests, specifically attending to power relations and challenging the foundations of knowledge construction and institutions of education (Habermas 1971; Kumagai 2014; Ng et al. 2015; Ng et al. 2019a). Thus, in the health professions, those drawn to RP have used it in relation to professional identity formation, in partnering with patients and communities, enhancing compassionate care and professional self-care, and increasingly to address social inequities (Kinsella et al. 2012; Thille et al. 2018). Certainly, there is growing engagement of AE in relation to health professions’ social roles (Tan et al. 2019), which could be a stepping stone to uniting AE and RP.

This reunion comes just in time. Within the realities of uncertain practice emphasized by the pandemic, practitioners were also called to act in response to social movements like Black Lives Matter. A combined AE and RP approach would potentially prepare professionals to respond effectively, compassionately, and equitably to future health and social crises and challenges. As health professions schools have begun to increasingly grapple with questions of equity and diversity, it may be that the capability to question and create knowledge in relation to power (Kinsella and Whiteford 2009) may become a focal point for AE scholars. In this way, RP could be positioned within an AE approach as a capability to continually question knowledge claims, their sources, and implications with explicit awareness of power relations. RP offers an epistemology of practice that continually represents the novelty, ambiguity, and uncertainty that AE aims to prepare learners for. For example, without this questioning, disability and rehabilitation topics are often taught in ways that perpetuate dominant discourses, with disability situated within the individual as opposed to disability as societally constructed, created, and perpetuated. Without critical reflection, this approach can actually further disable individuals through stigma and ableism. RP may offer an explicit imperative to question who best practices were developed by and for, who is excluded, and why, and how best practices might actually harm. An RP approach would necessitate seeking out connections to clients’ thoughts and feelings about their experiences living with a disability, and critically reflective practice would bring in critical knowledge and theory, such as critical disability studies, to challenge dominant or consensus-based definitions of “disability” and “rehabilitation.” These approaches are risky yet necessary; entire rehabilitation professions have been built upon these dominant discourses and yet there is an ethical and sustainability imperative to continue to question and change (Phelan 2011). Integrating RP approaches into AE in this way may be one mechanism for accentuating socially aware and compassionate care, where these possibilities may otherwise be occluded by medicine’s dominant orientation to objectivity and professionalism.

AE could also offer RP practical and tangible approaches to refining its theory through experimental research. Moon (1999) stated, “The fact that reflective practice seems to have become tied up with the essence of being a professional rather than the activity of facilitating learning or caring may have much to do with the manner in which the literature has built on Schön’s original work on the reflective practitioner as it also concerns itself with the professional aspects.” Indeed, criticisms of RP are extensive and include the lack of exploration of how it might improve learning and care. In contrast, ways of thinking about education that have advanced AE pedagogies include measurements that aim to assess the extent to which pedagogical approaches support the development of preparation for future learning (Bransford and Schwartz 1999). For example, many studies use measurements that require learners to learn and apply new information in order to generate a successful solution (Chaudhary et al. 2019; Mylopoulos and Woods 2014; Daniel L. Schwartz and Martin 2004; Steenhof et al. 2019, 2020). Recently, RP-focused scholars have borrowed this research design to study the impacts of different approaches to teaching for reflective capabilities and practices (Boyd et al. 2022; Ng et al. 2022). In doing so, the analytic approach needs to attend carefully to epistemological alignment and outcome assessment compatibility. Overall, these research designs historically used to study AE may afford an otherwise underdeveloped ability in health professions education research to carefully advance knowledge of how and why teaching for RP might work.

### Advancing curriculum and pedagogy

Some of the pedagogical strategies of AE may be challenging for learners to embrace against a backdrop of a biomedical culture that has historically valued objectivity and certainty; and here, perhaps RP could support learners in appreciating the learning process required for AE (Ng et al. 2019b). For example, to engage in productive failure, learners need to feel not only psychologically safe, but may also benefit from appreciating that navigating continual indeterminate zones of practice is what it means to be a professional. Here the strengths of RP in describing the essence of practice—that being a professional means navigating the swamps of practice continually—may bolster the pedagogical spaces required by AE. One of the reasons that RP has gained such popularity is that its description of learning from experience is so resonant with practicing professionals. Therefore, using the strengths of RP to help learners understand the essence of professional practice in fact behooves them to embrace and learn from failure and may prepare them to learn through the pedagogy of productive failure. Doing so would also require faculty to shift their role, opening up and demonstrating vulnerability, to a point, to represent their navigation of the messiness of practice as the norm.

Approaches to educating for AE may also complement and bolster RP. RP has been said to “flip the problem on its head,” wherein the messy and artistic aspects of practice are foregrounded, and the tidy and technical aspects of practice backgrounded. The practicality of such an endeavour becomes a key challenge. In current society, health professionals need to graduate with competencies. No one would want to undergo surgery led by a neurosurgeon who is capable of considering ethical dilemmas thoughtfully but has a poor understanding of neuroanatomy. While this is exactly the type of false dichotomy Schön and others argued against (Kinsella 2007) it is clear that RP’s pedagogical offshoots lack direction to integrate learning of, for example, neuroanatomy with learning how to recognize and respond to ethically important moments in practice. Here, the benefits of bridging with AE are clear. As noted previously (Ng et al. 2020), AE educational approaches to fostering health professions expertise emphasise conceptual knowledge development (knowing why) alongside the more traditional emphasis on procedural fluency (knowing how). The fluency of knowing what to do (how) enables navigation of routine problems of practice, while depth of understanding *why* allows a shift beyond formulaic applications of knowledge in practice when necessary. Being able to make this shift aligns with research on RP demonstrating that learners monitor their own practice and notice when it might be necessary to deviate from a checklist approach, whether in the case of an unexpected technical or ethical dilemma. Thus, there could be potential for the teaching of RP through the AE educational approaches used to integrate knowing why and how.

#### Back to the case/story

Let us return to the application of AE and RP in teaching interprofessional collaborative competencies in the case of Samuel. Faculty (potentially including patients as teachers) would share their first-hand accounts of the reality of long-term care, including its political and practical complexities. These stories would balance the very real challenges of practice in this context with stories of hope and impact. These stories help set the tone for an epistemology of practice—that knowledge and practice in this space is dynamic and complicated by the realities of social and political impacts on what is possible in long-term care. Interprofessional learners work to support Samuel. Personal and situational details about Samuel are shared, grounding the students’ learning in a focus on Samuel as a whole person. The student team must consider the complexity of physical and mental health conditions within the social context and plan for discharge. As they engage in the discharge planning process, they are provided with additional information on differing individual and family preferences regarding discharge. As the team members grapple with their own priorities, they are required to engage in dialogue about their dilemmas, a range of potential responses, and productive tensions. They also consider the many structural and systemic factors that may impact on team process. Learners then explore varied discharge plans and possible next steps to support the individual and family. Profession-specific procedural expertise must be communicated within a context where the science of collaboration and communication, inclusive of the patient and family, become driving factors in the process of collaborative decision-making. Opportunities for productive struggle followed by explicit instruction can be built into complex case descriptions that are not easily resolved. By adding questions like “what ifs…” as appropriate, experienced facilitators can use a critically reflective approach to guided discovery, to shift learner discussion to critically reflective dialogue, where they attend to assumptions, issues of power, and societal norms to foster fundamental changes in perspectives and practices.

## Conclusion

AE and RP are two large bodies of work originating in constructivism that clearly describe desirable professional practice. Both aim to prepare professionals who can do what is best for the patient and situation before them, drawing on extant knowledge and innovating when that knowledge reaches its limits in uncertain or complex aspects of practice. They have diverged over time, with AE building its body of constructivist knowledge and pedagogy and RP moving more toward humanistic and transformative paradigms of education, with increasing criticality. No single body of work is without its gaps. Bringing AE and RP back into explicit conversation with one another could be fruitful, enabling the amplification of strengths. Key to this conversation is thoughtfulness around paradigmatic alignment and integration. AE could offer RP practical and tangible approaches to curriculum design and pedagogical techniques that can be tested and enhanced through research designs common in the AE literature. It could also provide educators with more direct guidance and grounding in knowledge bases that ensure specific learning beyond the typical RP focus on the meaning of being a professional. RP may offer AE a mechanism beyond self-reflection, providing a constant reminder to hedge against believing knowledge or practice as immutable and with a focus on critically reflective practice, a reminder to generate and enact knowledge about power. It could also provide a practice epistemology framing, which we argue could enable an ongoing rich understanding of the realities of practice, such that pedagogies like productive failure could be more fully embraced by learners in high-performing lines of work. Together, AE and RP may overcome the age-old dilemma of “rigour versus relevance.” Schön wrote that the practitioner must decide whether to remain on the high-hard ground where technical rationality can solve routine problems, or venture into the “swampy lowlands” that Schön describes as indeterminate zones of practice (Schön 1983) where reflective practice (or adaptive expertise) would be required. But these are *not* the only choices to be made. Rather, by combining the strengths of AE and RP and their pedagogical descendants, the adaptive expert and reflective practitioner will be well-equipped to navigate the future of professional practice.

As Joe Kincheloe said, “We cannot simply attempt to cultivate the intellect without changing the unjust social context in which such minds operate. Critical educators cannot just work to change the social order without helping to educate a knowledgeable and skillful group of students. Creating a just, progressive, creative, and democratic society demands both dimensions of this pedagogical progress.” (Kincheloe 2004) Perhaps combining AE and RP offers us these two dimensions for the better development of knowledge, skill, and society.
